# Structure and characterization of a novel chicken biotin-binding protein A (BBP-A)

**DOI:** 10.1186/1472-6807-7-8

**Published:** 2007-03-07

**Authors:** Vesa P Hytönen, Juha AE Määttä, Einari A Niskanen, Juhani Huuskonen, Kaisa J Helttunen, Katrin K Halling, Henri R Nordlund, Kari Rissanen, Mark S Johnson, Tiina A Salminen, Markku S Kulomaa, Olli H Laitinen, Tomi T Airenne

**Affiliations:** 1 NanoScience Center, Department of Biological and Environmental Science, P.O. Box 35 (YAB), FI-40014 University of Jyväskylä, Finland; 2 NanoScience Center, Department of Chemistry, P.O. Box 35, FI-40014 University of Jyväskylä, Finland; 3 Institute of Medical Technology, FI-33014 University of Tampere, Finland; 4 Department of Biochemistry and Pharmacy, Åbo Akademi University, Tykistökatu 6 A, FI-20520, Turku, Finland; 5 A. I. Virtanen Institute, Department of Molecular Medicine, University of Kuopio, P.O. Box 1627, FI-70211 Kuopio, Finland; 6 Department of Materials, ETH Zürich, CH-8093 Zürich, Switzerland

## Abstract

**Background:**

The chicken genome contains a *BBP-A *gene showing similar characteristics to avidin family genes. In a previous study we reported that the *BBP-A *gene may encode a biotin-binding protein due to the high sequence similarity with chicken avidin, especially at regions encoding residues known to be located at the ligand-binding site of avidin.

**Results:**

Here, we expand the repertoire of known macromolecular biotin binders by reporting a novel biotin-binding protein A (BBP-A) from chicken. The BBP-A recombinant protein was expressed using two different expression systems and purified with affinity chromatography, biochemically characterized and two X-ray structures were solved – in complex with D-biotin (BTN) and in complex with D-biotin D-sulfoxide (BSO). The BBP-A protein binds free biotin with high, "streptavidin-like" affinity (K_d _~ 10^-13 ^M), which is about 50 times lower than that of chicken avidin. Surprisingly, the affinity of BBP-A for BSO is even higher than the affinity for BTN. Furthermore, the solved structures of the BBP-A – BTN and BBP-A – BSO complexes, which share the fold with the members of the avidin and lipocalin protein families, are extremely similar to each other.

**Conclusion:**

BBP-A is an avidin-like protein having a β-barrel fold and high affinity towards BTN. However, BBP-A differs from the other known members of the avidin protein family in thermal stability and immunological properties. BBP-A also has a unique ligand-binding property, the ability to bind BTN and BSO at comparable affinities. BBP-A may have use as a novel material in, e.g. modern bio(nano)technological applications.

## Background

Several biotin-binding proteins have been characterized from egg-laying vertebrates. The best known of these proteins is chicken avidin (AVD) [1-4]. This tetrameric ~60 kDa egg-white protein, together with its bacterial homologs streptavidin and bradavidin [5-7], has a fascinating feature, the ability to bind a small water soluble vitamin, D-biotin (BTN), with tremendous affinity (K_d _≈ 10^-15 ^M for AVD) [5]. The formation of the extraordinary strong protein-ligand complex is a result of "perfect" structural complementarity between the (strept)avidin ligand-binding site and BTN [8,9], and optimized "packing" of the overall tertiary and quaternary structure, too [5,10]. Although BTN is thought to be the natural ligand of (strept)avidin, these proteins are known to bind naturally-occurring as well as synthetic BTN analogous and their derivatives, which include the biotechnologically valuable 2-iminobiotin, 4'-hydroxyazobenzene-2-carboxylic acid (HABA) and desthiobiotin (for a review, see [1,5]). However, (strept)avidin has clearly weaker affinity for ligands other than BTN. The biological role of AVD is still partially unclear, but it has been postulated to function as an antimicrobial defence protein in chicken by ensuring that no free biotin is present in egg white; a vitamin required for the growth of bacteria [1]. Nevertheless, it is the numerous bio(nano)technological applications, where AVD's unique biotin-binding property has been utilized, which have made AVD one of the most well-known proteins.

In addition to AVD of chicken egg-white, other proteins capable of binding BTN tightly, biotin-binding protein I (BBP-I) [11-13] and II (BBP-II) [14,15] of chicken egg-yolk, have been reported. Nothing is known about the affinity of BBP-I/II for ligands other than BTN. Although immunologically similar and having comparable N-terminal sequences [15,16], BBP-I and BBP-II differ in their thermal stability, BBP-I being more stable than BBP-II [14]. The biological functions of the two BBP forms are only partly resolved, but they are known to be synthesized in the liver and believed to transport BTN via plasma to the egg-yolk [14,16,17]. More specifically, it is believed that BBP-I has a general role as a transport protein in hen plasma, whereas BBP-II may be needed for the efficient deposition of BTN into the yolk of maturating oocytes [14].

There is a substantial evidence that the quaternary structure of BBP-I/II is tetrameric, but it is not clear whether the tetramers are formed as a result of assembling four monomers or if the tetramers result from limited proteolysis of a single polypeptide containing all the binding sites [13,16]. Evidence for the former hypothesis became available when the putative genes and cDNAs encoding the BBP-I/II proteins were reported in 2005 by Niskanen and co-workers [18]: the exon-intron structures of the BBP genes [18], named as *BBP-A *and *BBP-B*, mimic those of *AVD *and avidin-related genes (*AVR*s) [19-21] and they encode proteins with a single ligand binding site per polypeptide chain. It is worth mentioning that it is not yet completely clear whether the *BBP-A *and *BBP-B *genes really encode for either of the earlier characterized BBP-I and BBP-II proteins; *BBP-A *in particular seems to encode a novel protein, BBP-A, not characterized before [18].

In the present study, we show that the chicken *BBP-A *gene encodes a functional homotetrameric protein. We report two different X-ray structures of BBP-A, one in complex with BTN and an other one in complex with D-biotin D-sulfoxide (BSO), at 2.1 Å and 1.75 Å resolution, respectively. To our knowledge, no other structures of any proteins in complex with BSO have been reported before. Using several biochemical methods, we show that BBP-A binds BSO even tighter than BTN. A comparative study of the ligand-binding and physicochemical properties of BBP-A and AVD are presented together with data from site-directed mutagenesis.

## Results

### Biochemical characterization of BBP-A

BBP-A, expressed both in *E. coli *(bBBP-A) and in insect cells (iBBP-A), was isolated and purified using 2-iminobiotin affinity chromatography. A typical yield was 5 mg of pure protein per one litre of culture medium for both expression systems. The mutations A74S and T118F, which were made in order to study the differences in the molecular origin of the biotin-binding affinity and the thermal stability of BBP-A as compared to chicken AVD, had no significant effect on the protein yields. The isolated proteins were shown to be over 95% pure using SDS-PAGE analysis (data not shown). A molecular weight of 13999.0 Da was determined for the expressed bBBP-A using mass spectrometry, and it matched the molecular weight calculated from the expression construct.

**Figure 1:**
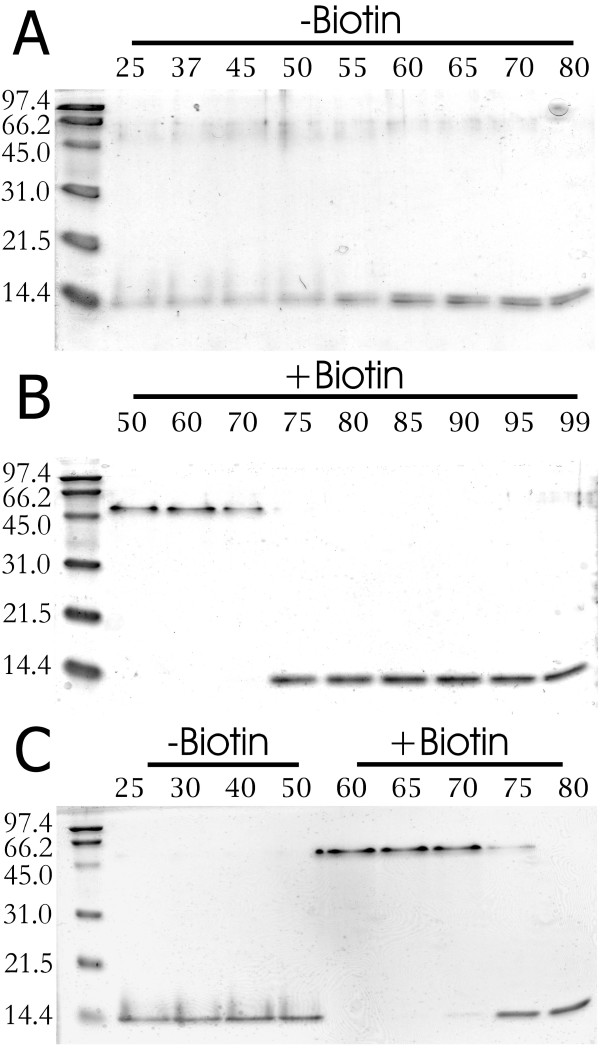
**SDS-PAGE based analysis of the thermal stability of the tetramers of BBP-A**. Samples saturated with BTN (+Biotin) prior to analysis as well as samples without added BTN (-Biotin) are shown. (a) bBBP-A, (b) bBBP-A in the presence of BTN and (c) bBBP-A(A74S) in the absence and presence of BTN. After acetylation in vitro, the samples were subjected to thermal treatment in SDS-PAGE sample buffer containing 2-mercaptoethanol and SDS. The different temperatures used in the analysis are indicated in the upper part of the figure (°C). The molecular weights of the standard proteins (Bio-Rad) are shown on the left.

The oligomeric state of bBBP-A, bBBP-A(A74S), bBBP-A(T118F), iBBP-A, bAVD and wtAVD in solution was analysed using gel filtration chromatography. All of the proteins eluted as single peaks in this analysis, demonstrating the homogeneity of the samples. The apparent molecular weights of iBBP-A (60.8 kDa) and wtAVD (62.5 kDa), based on gel filtration analysis, corresponded well with the theoretical molecular weights for their respective tetramers. As expected, the proteins produced in bacterial cells showed apparent molecular weights lower than the corresponding proteins expressed in insect cells, at least partially due to the absence of glycosylation (bBBP-A, 42.7 kDa and bAVD, 55.3 kDa). The mutations A74S and T118F had no effect on the appearance of BBP-A in the gel filtration analysis. Nor did the addition of BTN to the samples significantly alter the apparent molecular masses of the analysed proteins. This, together with the results showing that BTN significantly stabilizes tetramers of bBBP-A (see below; Table 1 and Figure 1), supports the idea that the quaternary structure of bBBP-A is tetrameric, an idea that is also supported by the crystal structures of bBBP-A – BSO and bBBP-A – BTN (see below).

**Table 1:** Biochemical properties of different BBP-A forms.

	**Gel filtration**				**SDS-PAGE-based thermostability assay**		**Dissociation of fluorescent BTN (25°C)**		**DSC**	
	Elution time (min)		Molecular mass (kDa)^a^		T_r _(°C)		k_diss _(10^-5 ^s^-1^)	Release 1 h (%)	T_m _(°C)	
	-BTN	+BTN	-BTN	+BTN	-BTN	+BTN			-BTN	+BTN
bBBP-A	29.9	30.1	42.7	40.9	N. D.	70	54.8	86	N. A.	103.4
bBBP-A (A74S)	30.0	30.1	41.9	40.8	N. D.	75	47.1	83	N. A.	103.2
bBBP-A (T118F)	30.1	29.9	41.3	42.7	N. D.	70	69.1	94	N. A.	102.3
iBBP-A	28.4	28.4	60.8	59.7	N. D.	75	20.4	53	N. M.	N. M.
AVD	28.2	28.2	62.5	63.6	60^b^	90^b^	2.0	10	83.5^c^	117.0
bAVD	28.8	28.8	55.3	55.1	60^b^	90^b^	1.1	5	N. M.	N. M.

N. D. Protein appeared mainly in monomeric form already at room temperature; N. A. Not applicable; N. M. Not measured.

^a^Molecular mass calculated from the measured elution times of thyroglobulin (670 kDa), gamma-globulin (158 kDa), BSA (67 kDa), ovalbumin (44 kDa) and myoglobin (17 kDa).

^b^Results from ref. [30].

^c^Results from ref. [33].

**Figure 2:**
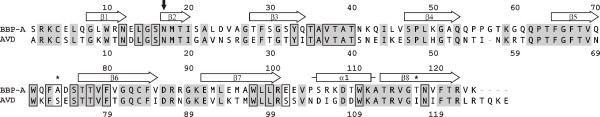
**Sequence alignment of BBP-A and AVD**. Identical residues are shown in gray shading. The signal peptides are omitted from the alignment. Secondary structure elements are indicated according to the BBP-A – BSO structure (chain A). The amino acids within 4 Å of BSO in the BBP-A – BSO structure (and the equivalent residues in AVD) are boxed. The residues mutated in this study are indicated by an asterisk. The glycosylation site of BBP-A is indicated by an arrow.

**Figure 3:**
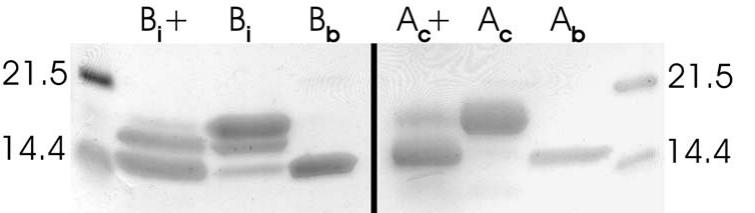
**Deglycosylation analysis of BBP-A**. The BBP-A produced in insect cells was treated with Endo H_f _glycosidase (B_i_+) and analysed with SDS-PAGE. Untreated control sample (B_i_) and BBP-A produced in bacteria (B_b_) were also analysed. For comparison, the same analysis was performed for AVD isolated from chicken (A_c_+). Chicken AVD control sample (A_c_) as well as AVD produced in bacteria (A_b_) [30] are also shown. The molecular weight markers are shown on both sides of the gel (14.4 and 21.5 kDa).

The amino acid sequence of BBP-A contains one putative N-glycosylation site (Figure 2), which corresponds to an N-glycosylation site known to be present and glycosylated in AVD [1]. To see whether iBBP-A was also glycosylated, the protein was treated with Endoglycosidase H and analysed on SDS-PAGE (Figure 3). After the deglycosylation treatment, the iBBP-A protein had a molecular weight comparable to that of bBBP-A, a molecular weight clearly lower than the molecular weight of the untreated iBBP-A sample. This result, together with the control data (wtAVD), indicates that the N-glycosylation site of BBP-A was indeed glycosylated in insect cells and the size of the attached carbohydrate is comparable to that present on wtAVD.

**Figure 4:**
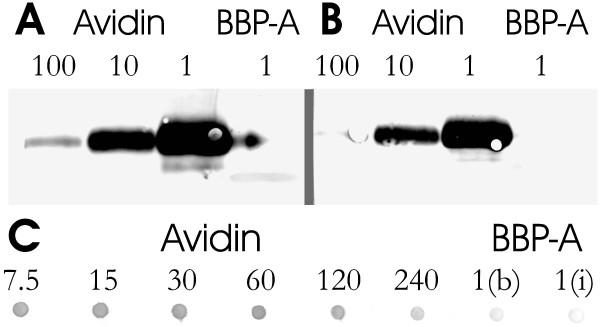
**Immunological cross-reactivity of chicken BBP-A and AVD**. (a) Western blot analysis of 1/100, 1/10 and 1/1 diluted samples of chicken AVD (10 μg) and undiluted bBBP-A protein (10 μg). The polyclonal antibody TdaVII against AVD [51] was used as the primary antibody. (b) The same analysis as in A using another polyclonal antibody (University of Oulu, Finland). (c) Dot-blot analysis of BBP-A (10 μg) produced in bacteria 'b' and in insect cells 'i'. The polyclonal antibody TdaVII against AVD was used as the primary antibody. Serial dilutions of AVD (10 μg) were used as controls (the dilution factors are indicated in the figure).

The immunological cross-reactivity of BBP-A and wtAVD was studied with two polyclonal antibodies made against chicken AVD (Figure 4). Neither of the antibodies did cross-react with BBP-A in a Western blotting analysis. The reactivity of both antibodies was significantly weaker with iBBP-A (10 μg) than in the case of a 100-fold dilution of wtAVD (0.1 μg). In dot-blot analysis, only one antibody, the TdaVIII antibody, was used to test whether it detects iBBP-A and bBBP-A. In agreement with the immunoblot analysis, no or only negligible cross-reactivity was observed.

### Stability of the tetrameric assembly of BBP-A

The thermal stability of the tetrameric form of BBP-A was analysed in the presence of 2-mercaptoethanol using an SDS-PAGE-based method described in [22]. Both bBBP-A and iBBP-A appeared mainly in monomeric forms on SDS-PAGE gels already at room temperature in the absence of BTN, whereas addition of BTN stabilised the tetrameric form, which was stable until the temperature was raised to 70°C (Table 1 and Figure 1). In the presence of BTN, glycosylated iBBP-A had slightly better thermal stability (T_r _= 75°C) compared to bBBP-A (T_r _= 70°C). Both bBBP-A and iBBP-A were clearly more thermally labile than wtAVD. The mutations A74S and T118F had no significant effect on the stability of the tetrameric forms of bBBP-A (Table 1, Figure 1).

Differential scanning calorimetry (DSC) was not applicable to the bBBP-A forms in the absence of ligands, since a clear, single denaturation temperature could not be measured for these proteins. For bBBP-A, two peaks at around 51°C and 68°C were obtained. In the presence of BTN, however, the value of T_m _was around 100°C for all bBBP-A forms. These values are significantly lower in comparison to those measured for wtAVD (Table 1). Furthermore, the thermal stability of bBBP-A and AVD was analysed in the presence of BSO and D-biotin sulfone (Table 2). Only negligible differences were, however, found between the effects of these two ligands and BTN on the thermal stabilities of bBBP-A and AVD.

**Table 2:** Dissociation of BTN and its oxidized forms from BBP-A and AVD. Fluorescence spectroscopy and radiobiotin dissociation analysis data at 40°C are shown. Binding enthalpies were measured by ITC at 25°C. bBBP-A and commercial chicken AVD was used in the analyses.

Ligand	k_diss _× 10^-4 ^s^-1^	Enthalpy (kcal/mol) (ITC)		T_m _(°C) (DSC)	
	BBP-A	AVD	BBP-A	AVD	BBP-A

BSO	1.3^a^	-29.3 ± 0.1	-25.9 ± 0.1	116.9 ± 0.2	100.4 ± 0.2
D-biotin sulfone	3.3^a^	-25.1 ± 0.1	-21.4 ± 0.1	117.0 ± 0.0	101.4 ± 0.2
BTN	5.4^b ^(4.4^c^)	-22.6 ± 0.1	-20.5 ± 0.1	117.0 ± 0.7	103.4 ± 0.1

^a^From fluorescence spectroscopic analysis, excess of BTN was used as a competitive ligand.

^b^From fluorescence spectroscopic analysis, excess of BSO was used as a competitive ligand.

^c^Value obtained from [^3^H]-biotin dissociation analysis, excess of BTN was used as a competitive ligand.

### The overall X-ray structures of BBP-A

**Figure 5:**
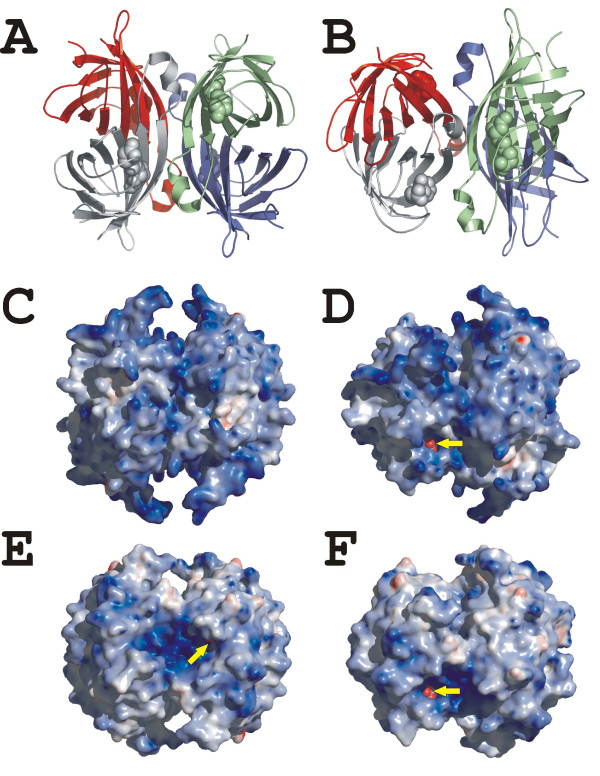
**The overall X-ray structure of chicken BBP-A**. (a, b) A tetrameric ribbon model of the BBP-A – BSO structure. (c, d) Electrostatic potentials mapped onto the molecular surface of the BBP-A – BSO structure. (e, f) Electrostatic potentials mapped onto the molecular surface of the AVD [PDB: 2AVI] [23] structure after superimposition on the BBP-A structure. The views shown in (a), (c) and (e) are rotated 45° around the *X*-axis in (b), (d) and (f). The BSO and BTN ligands of the BBP-A – BSO and AVD structures, respectively, are shown as spheres. The yellow arrows pinpoint visible parts of the valeric acid tails of BSO and BTN seen in (d), (e) and (f).

The crystal structures of bBBP-A in complex with BTN and BSO were solved at 2.1 Å and 1.75 Å resolution, respectively. The statistics for the structure determinations are summarized in Table 3. The overall BBP-A – BTN and BBP-A – BSO structures are practically identical to each other; the Cα atoms of these structures superimpose with an *rmsd *of < 0.2 Å. The BBP-A structures consist of four identical subunits, each subunit adopting a β-barrel fold of eight anti-parallel β-strands with an α-helix positioned between the β7- and β8-strands (Figure 5a,b). The BTN/BSO-binding site is located at the open end of each β-barrel. The overall fold of BBP-A is similar to that seen for the known structures of the AVD family complexes, i.e. streptavidin-BTN [8], AVD-BTN [9,23], AVR2-BTN [24] and AVR4-BTN [25] complexes. The solvent accessible surface area is, however, over 10% larger in the BBP-A structures (> 21000 Å^2^) compared to the AVD-BTN structures (< 19000 Å^2 ^[PDB: 1AVD and 2AVI]). The electrostatic surface properties of BBP-A and AVD differ, too, BBP-A having a seemingly more positively charged surface than AVD (Figure 5c–f).

**Table 3:** Data collection and structure determination statistics for BBP-A

**Data collection**^a^	**BBP-A – BTN**	**BBP-A – BSO**
Wavelength (Å)	1.063	1.063
Beamline	I711 (MAX-lab)	I711 (MAX-lab)
Detector	MarCCD 165	MarCCD 165
Resolution (Å)	25-2.1 (2.2-2.1)	25-1.75 (1.85-1.75)
Unique observations	10571 (1351)	26447 (3993)
I/sigma	14.06 (4.31)	12.78 (3.24)
*R*_factor _(%)^b^	10.5 (48.6)	7.7 (48.2)
Completeness	99.9 (100)	99.9 (100)
Redundancy	8.6 (8.7)	4.8 (4.8)

**Refinement**		

Space group	*I*4_1_22	*P*2_1_2_1_2
Unit cell:		
a, b, c (Å)	61.7, 61,7, 179.7	79.7, 56.0, 57.1
α, β, γ (°)	90, 90, 90	90, 90, 90
Monomers (asymmetric unit)	1	2
Resolution (Å)	25-2.1	25-1.75
*R*_work _(%)	19.5	19.3
*R*_free _(%)	22.0	23.3
Protein atoms	969	1965
Heterogen atoms	22	34
Solvent atoms	61	212
*R.m.s.d:*		
Bond lengths (Å)	0.014	0.014
Bond angles (°)	1.6	1.6
Ramachandran plot:		
Residues in most favored regions	93.3	93.3
Residues in additional allowed regions	6.7	6.7
Residues in generously allowed regions	0	0
Residues in disallowed regions	0	0

^a^The numbers in parenthesis refer to the highest resolution bin.

^b^Observed *R*-factor from XDS [53].

### Mode of BTN and BSO binding

**Figure 6:**
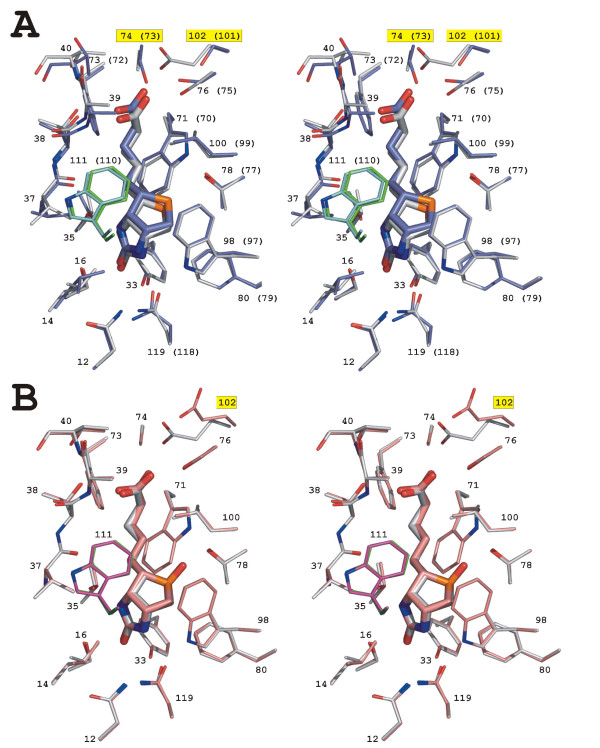
**The binding mode of BTN and BSO**. (a) BTN (thick sticks) and the amino acids within 4 Å of BTN in the BBP-A – BTN and AVD [PDB: 2AVI] structures are shown. The carbon atoms of residues from subunit 1 of the BBP-A and AVD structures are coloured light grey and blue, respectively. The carbon atoms of Trp111 (Trp110) from subunit 2 of BBP-A (AVD) are coloured green (cyan). The amino acids of BBP-A (AVD) are numbered. The labels of the non-conserved residues are indicated with a yellow background. (b) BTN and BSO (thick sticks), and the amino acids within 4 Å of the ligands in the BBP-A – BTN and BBP-A – BSO structures are shown. The carbon atoms of residues from subunits 1 of the BBP-A – BTN and BBP-A – BSO structures are coloured light grey and light red, respectively. The carbon atoms of Trp111 from subunit 2 of the BBP-A – BTN and BBP-A – BSO structure are coloured green and dark red, respectively. The amino acids are numbered. Glu102, whose side chain has a different conformation in the BBP-A – BTN and BBP-A – BSO structures, is labelled with a yellow background.

The biotin-binding site of the BBP-A – BTN structure mimics the biotin-binding site of AVD [9,23]. The amino acid residues within 4 Å of BTN in the complex with BBP-A (Asn12, Leu14, Ser16, Tyr33, Thr35, Val37, Thr38, Ala39, Thr40, Trp71, Phe73, Ala74, Ser76, Thr78, Phe80, Trp98, Leu100, Glu102, Asn119 and Trp111) are not only highly conserved but also have conformations similar to those seen in AVD (Figure 6a). Actually, only one polar (Glu102) and one hydrophobic residue (Ala74) within 4 Å of BTN are not conserved between the BBP-A – BTN and AVD-BTN complex [PDB: 2AVI] structures. The side-chain oxygen of Glu102 of BBP-A is hydrogen bonded to one of the valeric oxygen atoms of BTN, whereas in AVD the equivalent residue, Ser101, can not form a hydrogen bond to the valeric oxygen because the distance between the two atoms is too long (5.8 Å). The methyl carbon of Ala74 of BBP-A, on the other hand, is positioned only 3.7 Å away from the valeric O10B atom of BTN (numbering according to [26]), but is not likely to be hydrogen bonded with BTN unlike the side-chain oxygen atom of the corresponding Ser73 in AVD, which is hydrogen bonded to BTN.

**Figure 7:**
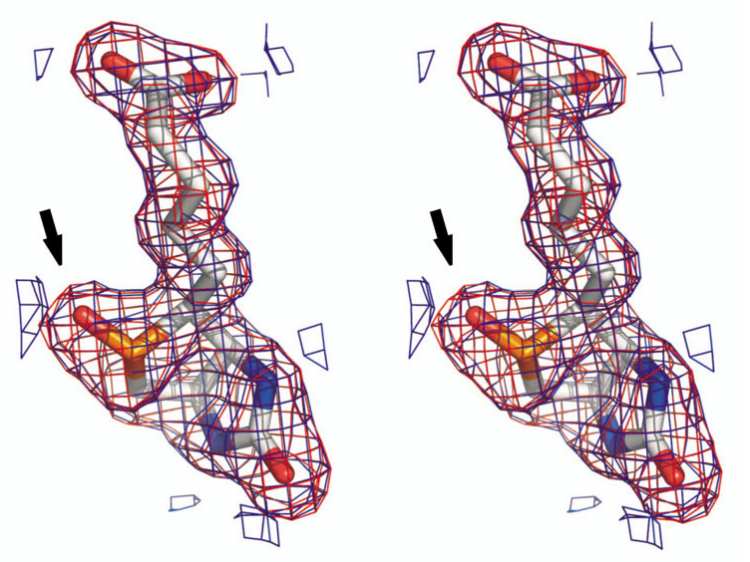
**Stereo image of electron density around the BSO ligand of the BBP-A – BSO structure**. The additional oxygen atom on the BSO ligand (in comparison to BTN) is indicated by an arrow. The 2Fo-Fc (blue) and Fo – Fc (red) maps calculated without the ligand but drawn around BSO of the final model are shown at the level of 1.0 and 3.0 sigma, respectively.

In addition to the BBP-A – BTN complex structure, we solved the X-ray structure of BBP-A – BSO, too. To our knowledge, BSO is not found in any other protein structure presently within the Protein Data Bank (PDB) [27]. Electron density for the BSO ligand was clearly seen in the difference map of the BBP-A – BSO structure (Figure 7). The binding mode of BSO to BBP-A is surprisingly similar to that of BTN (Figure 6b) despite the additional oxygen atom that is covalently linked to the sulphur atom of the bicyclic ring system of BSO but is missing from BTN. The distance between the sulfur atom of BTN and the OG1 atom of Thr78 (3.4 Å), for example, is very close to the BSO – Thr78 distance (3.6 Å). In the known AVD-BTN complex structures [PDB: 2AVI and 1AVD], the corresponding distances are 3.2 Å and 3.8 Å. The only noticeable difference around the ligands in the two BBP-A structures was found at Glu102, which in the BBP-A – BTN structure is hydrogen bonded to the valeric O10A atom of BTN but in the BBP-A – BSO structure was observed either to form a salt bridge with Arg115 (chains B and D) or to hydrogen bond to a structural water molecule (chains A and C). The difference in the conformation of Glu102 is not likely to result from the varying crystal contacts of the two BBP-A structures that crystallized as different space groups (data not shown). Moreover, the overall conformations of the BTN and BSO ligands bound to BBP-A were very similar, too.

### Subunit interfaces of BBP-A

Comparing BBP-A with AVD, we saw differences at subunit interfaces 1–3 and 1–4, whereas the amino acid residues found at the 1–2 interface were conserved and had similar conformations (numbering of subunit interfaces according to [9]).

**Figure 8:**
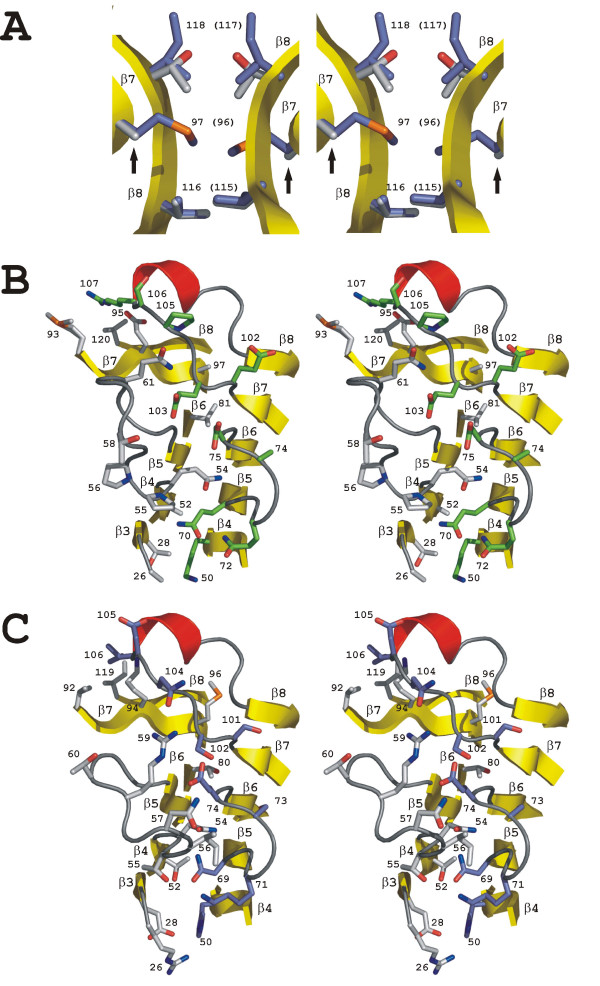
**Stereo images of the 1–3 and 1–4 subunit interfaces of BBP-A and AVD**. (a) The 1–3 subunit interface of the AVD [PDB: 1VYO] [68] structure superimposed with the corresponding interface of the BBP-A – BSO structure. The carbon atoms of amino acids from BBP-A and AVD are coloured light grey and blue, respectively. Amino acids and β-sheets are numbered. Two occurrences of Ala97 in the BBP-A structure are indicated by arrows. (b, c) A simplified view of the 1–4 interface of the BBP-A – BSO (b) and AVD [PDB: 2AVI] [9] (c) structure after superimposition. The atoms of the non-conserved side chains of amino acids within 4 Å of subunit 1 of either the BBP-A or AVD structure are shown. For clarity, Met96 and Thr110 of BBP-A as well as the equivalent residues of AVD (Thr95 and Asp109) are not shown, and only one half of the symmetrical 1–4 interface is shown. The carbon atoms of residues from subunit 1 and 4 are respectively coloured light grey and green in BBP-A, and grey and blue in AVD. Amino acids and β-sheets are numbered.

Of the three residues (Val116, Gly117 and Thr118) within 5 Å from each other at the 1–3 subunit interface of the BBP-A – BSO structure (Figure 8a), valine and glycine are conserved between BBP-A and AVD, while Thr118 is replaced by Ile117 in the AVD structure. In BBP-A there is no interaction equivalent to the hydrophobic or van der Waals interactions introduced by the two Met96 residues of AVD at the 1–3 subunit interface: in BBP-A, Met96 is replaced by Ala97 and the distance between Ala97 in the two subunits is about 11 Å. Generally, the 1–3 subunit interface seems to be more loosely packed in BBP-A compared to AVD, AVR4 [25] and even AVR2 [24] (data not shown).

The total contact surface area of the 1–4 interface of BBP-A – BSO is 2428 Å^2^, whereas the corresponding contact surface area in AVD is 2319 Å^2^. Twenty-six of the 49 amino acid residues found at the 1–4 interface within a contact radius of 4 Å are different between the BBP-A – BSO and AVD-BTN [PDB: 2AVI] structures. Hydrophobic residues located at the core of the 1–4 subunit interfaces are well conserved in BBP-A and AVD, whereas several differences among the polar residues were observed, especially at the interacting loop regions (Figure 8b,c). The hydrogen bond found in AVD between the OE2 atom of Glu28 of the β3 sheet and the NE2 nitrogen of His50 of the β4 sheet, for example, is not seen in BBP-A where Glu28 and His50 are respectively replaced by Thr28 and Lys50. The hydrogen bond formed between the side-chain atoms of Gln61 (L4,5 loop) and Glu103 (L7,8 loop) in BBP-A is, in turn, missing from AVD, where the residues are replaced by Thr60 and Ser102. Yet another example of the differences at the 1–4 subunit region between BBP-A and AVD was seen for the only α-helix and the β7-strand of these structures: in BBP-A the side-chain oxygen atom of Glu95 can form hydrogen bonds with the NE and NH2 atoms of the side chain of Arg107, whereas the atoms of the respective residues, Lys94 and Ile106, of AVD may introduce only van der Waals contacts. These and other differences (Figure 8b,c) reflect the varying architecture of the 1–4 interfaces in BBP-A and AVD.

### Ligand-binding analyses

**Figure 9:**
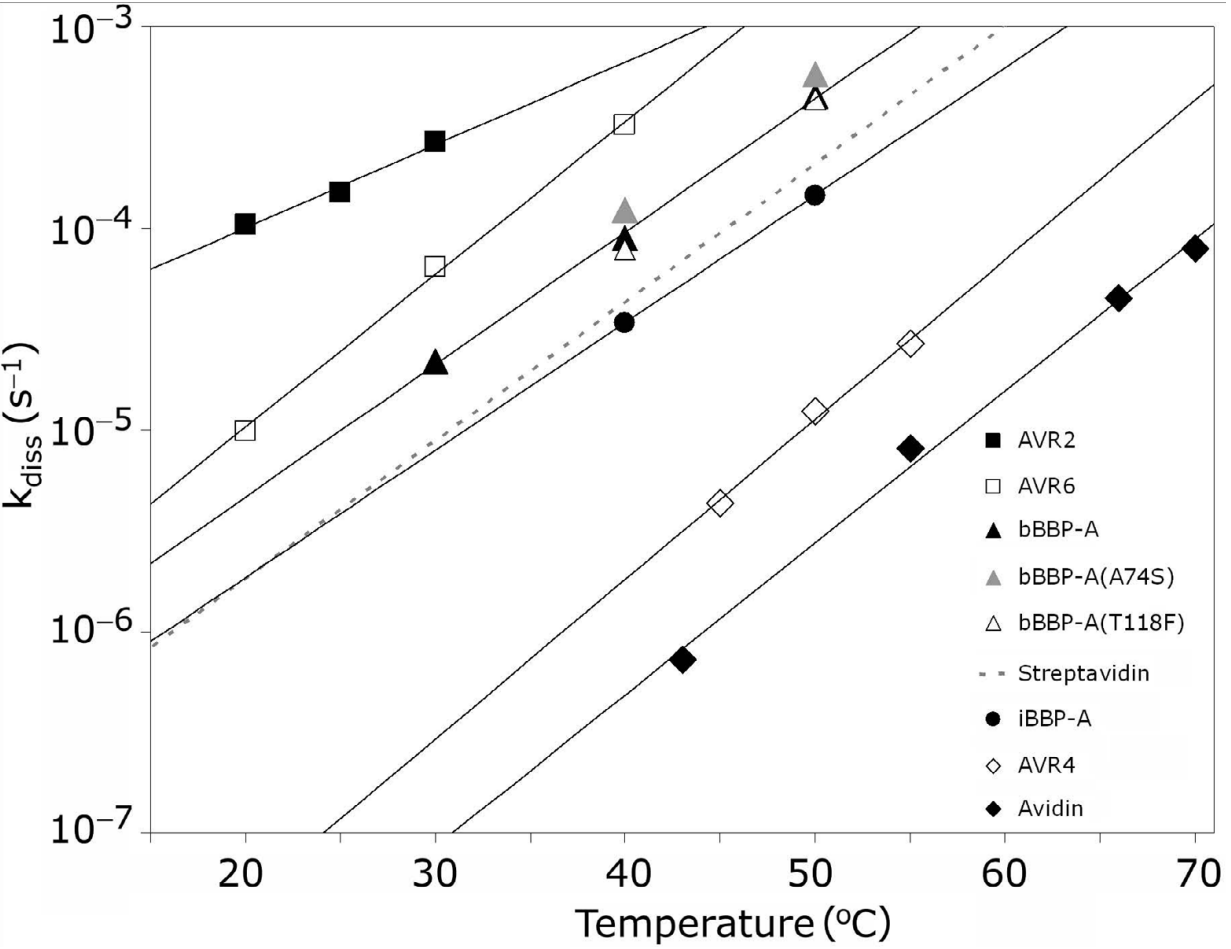
**Biotin dissociation analysis**. The dissociation of [^3^H]biotin from various proteins was determined over time. The individual dissociation rates of [^3^H]biotin measured for bBBP-A (black triangles), bBBP-A(A74S) (grey triangles), bBBP-A(T118F) (white triangles) and iBBP-A (black circles) are shown. Solid lines represent linear fit to data sets. Data measured previously for AVR2 (black rectangles), AVR6 (white rectangles) and AVR4 (white tilted rectangles), all produced in bacteria, are also shown [24, 28]. Data measured for chicken AVD (tilted black rectangles) are presented, too [42]. AVR6 and AVR4 carried the mutations C58S and C122S, respectively, which prevented the formation of intersubunit disulphide bridges [33]. The dissociation rates for streptavidin are shown as a dotted line according to [29].

The dissociation rate of [^3^H]biotin from BBP-A was measured using a competitive dissociation assay (Figure 9; Table 2). The analysis showed that bBBP-A has a dissociation rate about 100-fold higher than that of AVD [28], indicating that the affinity for BTN is lower in bBBP-A compared to AVD, but similar to that of streptavidin [29]. The dissociation rate of the fluorescent BTN conjugate ArcDia™ BF560-biotin was, in turn, about 30-fold higher for bBBP-A compared to wtAVD (Table 1). Interestingly, iBBP-A had an almost three-fold slower dissociation rate compared to bBBP-A. WtAVD showed a higher dissociation rate compared to the bAVD form produced in *E. coli*, which is in line with previously reported observations [30]. The A74S and T118F mutations of BBP-A did not significantly affect the biotin-binding properties of BBP-A (Table 1, Figure 9).

**Figure 10:**
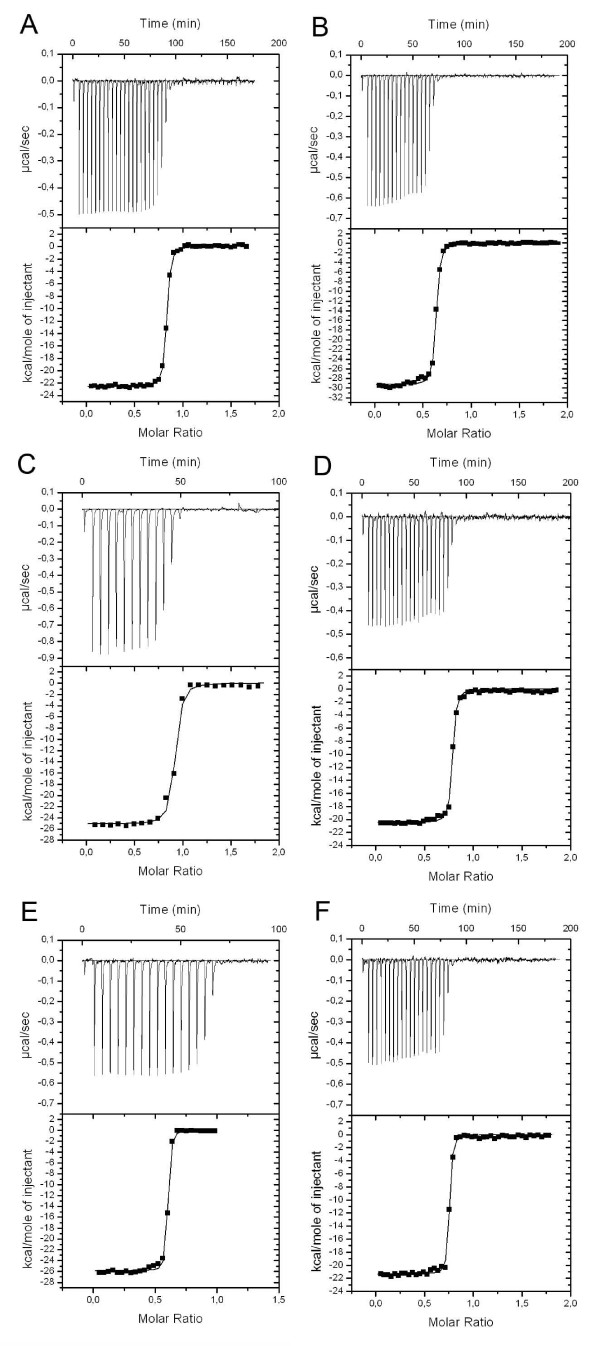
**Isothermal titration calorimetry analysis of bBBP-A and AVD**. Binding thermograms measured for (a) AVD-BTN, (b) AVD-BSO, (c) AVD – D-biotin sulfone, (d) bBBP-A – BTN, (e) bBBP-A – BSO and (f) bBBP-A – D-biotin sulfone complexes are shown at the upper part of the graphs. Non-linear least square curves (at the lower part of each graph) were fitted to enthalpies integrated from the individual titrations. For each titration, 0.5 mM ligand solution was used. The reaction volume was 10 μl except for the first titration, which was done in 2 μl.

The biotin-binding characteristics of bBBP-A and wtAVD studied by ITC analysis were similar for both proteins. Because the binding of BTN was tight (K_d _< 10^-9 ^M) in both cases, the binding constants could not be determined directly from the ITC data (Figure 10). The binding enthalpy could, however, be accurately determined, and the obtained values are listed in Table 2. In addition to BTN, the binding of BSO and D-biotin sulfone to wtAVD and bBBP-A were analysed using ITC (Table 2). The oxidised BTN forms exhibited a slightly higher binding enthalpy compared to BTN for both proteins studied. Overall, ligand binding to AVD released more heat, but a rather similar variation in the binding enthalpies of the studied ligands was observed in BBP-A and AVD. Therefore, the ITC analysis of the enthalpy of binding does not suggest different ligand preferences for BBP-A and AVD.

**Figure 11:**
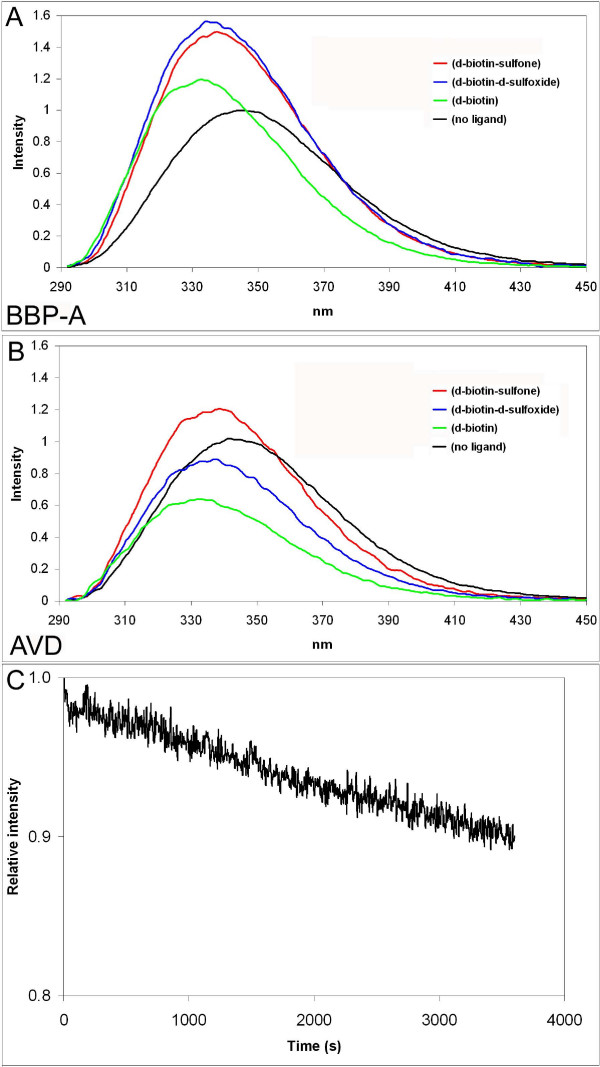
**Fluorescence spectroscopy analysis of BBP-A and AVD**. (a) Emission spectra of BBP-A in the absence of ligand (black) and with BTN (green), BSO (blue) or D-biotin sulfone (red) are shown. (b) Emission spectra of AVD. (c) Dissociation of D-biotin sulfone from BBP-A displaced with excess of BTN at 40°C. The measured emission intensity is plotted over time and corrected using the emission decay data from control measurements, which were made for protein saturated with BTN. Excitation at 280 nm was used.

The ligand-induced changes to the emission spectra of bBBP-A and wtAVD were studied, too (Figure 11). All biotin forms, BTN, BSO and D-biotin sulfone, enhanced the emission intensity of BBP-A. In contrast, only D-biotin sulfone increased the emission intensity of AVD, whereas BSO and especially BTN decreased the emission intensity of AVD. Compared to the ligand-free proteins, for each of the ligand-protein combinations the maximum in the emission spectra moved towards the shorter blue wavelengths. Altogether, these results indicate differences in the ligand-protein interactions between BBP-A and AVD, particularly reflecting variation in the local environment of tryptophan residues [31].

### EST database search

Chicken EST databases were searched using NCBI blastn [32] and the BBP-A and AVD cDNAs as query sequences. The search resulted in 9 significant (E-value < 1 × 10^-100 ^and score > 400) hits in the case of BBP-A, whereas the cDNA of AVD yielded 94 hits. Further analysis of the BBP-A hits showed that they correspond to mRNAs isolated from kidney and the adrenal glands (3 hits), the trunks of chicken stage 36 embryos (4 hits) and multiple-tissue preparations (2 hits). The AVD hits, in turn, corresponded to mRNAs isolated from lymphoid tissues (1), intestine (1), ovary (2), the chondrocytes of cartilage (6), multiple-tissue preparations (4), splenic T cells (2), pituitary gland, hypothalamus and pineal gland (1), and PBL macrophages (77).

## Discussion

Here, we report the biochemical and structural characterization of BBP-A, produced efficiently both in *E. coli *and *Spodoptera frugiperda *cells. This study demonstrates that BBP-A can be classified as a new member of the AVD family: BBP-A binds BTN with high affinity like all the other known members of the family [4,24,33] and has the β-barrel fold characteristic of AVD and of the more heterogeneous calycin superfamily of proteins [34]. The binding mode of BTN to BBP-A (Figure 6) is also highly similar to that observed in the known structures of AVD [9,23], AVR2 [24] and AVR4 [25]. The BBP-A protein is, however, biochemically and structurally clearly distinguishable from chicken AVD and the AVRs [1,4,18,24,25]. There are also differences in the immunological properties of BBP-A and AVD, since immunoblot and dot-blot analyses with polyclonal AVD antibodies did not show cross-reactivity between BBP-A and AVD (Figure 4).

Does BBP-A represent the earlier reported BBPs, BBP-I or BBPII? Based on the biochemical characterization of BBP-I [11,35-37], and BBP-II [14,15], this seems not to be the case. The [^3^H]-biotin dissociation rate constant of BBP-I reported in 1978 by Meslar and co-workers [11] is 2–4 times higher than that determined for bBBP-A and even 5–12 times higher in comparison to the glycosylated iBBP-A, which has a dissociation rate of [^3^H]biotin (Figure 9) in the same range as reported for streptavidin [29]. The pI (4.6) of the BBP-I protein differs from that calculated for BBP-A (pI = 9.75), too. Moreover, the reported N-terminal sequences of BBP-I and BBP-II do not match the sequence of BBP-A [18]. In conclusion, the BBP-A protein reported here seems to be a novel protein not described in any previous publications.

In order to study the molecular details of ligand recognition by BBP-A and compare the ligand-binding properties of BBP-A with the previously determined structures of AVD [9], streptavidin [8], AVR4 [25] and AVR2 [24], we crystallized bBBP-A in complex with BTN. Surprisingly, in one of the two BBP-A crystals that were analyzed, BSO was found bound to BBP-A even though only BTN was added in the co-crystallisation experiments. To our knowledge, this complex is unique, as no other protein structure has been reported in complex with BSO. What can be the source of the bound BSO? Based on the crystallization conditions (see Methods), it is not easy to say why one structure bound BTN and the other BSO. It is known that some BTN preparations may carry minor amounts of BSO [38], but based on mass spectrometry analysis (data not shown) no detectable amounts of BSO were found in the diluted BTN solution that was used for crystallization. One possibility is that the source of the BSO ligand seen in the BBP-A – BSO structure is BTN that had undergone oxidation during crystallization. Another possibility is that BBP-A had itself converted BTN to BSO by some yet unknown catalytic mechanism, but experimental proof for this hypothesis is lacking and the final explanation for the presence of BSO in one of the BBP-A structures remains to be studied.

The BBP-A – BSO structure inspired us to investigate whether BBP-A recognizes BSO with altered affinity in comparison to BTN. We analysed the binding of the fully oxidised sulfone form of BTN, too. To our surprise, the dissociation rate of BSO (fluorometric analysis at 40°C, Table 2) from BBP-A was similar or even slower than the dissociation rate of BTN (fluorometric and radiobiotin analysis at 40°C), whereas in the case of AVD the dissociation rate of BSO was more than 20 times higher in comparison to BTN (for AVD, the dissociation rate of BSO was determined using fluorometric analysis at 50°C (data not shown), whereas the dissociation of BTN was determined using radiobiotin assay at 50°C (Figure 9)).

This is the first reported case, to our knowledge, where the product of a naturally occurring gene encoding a BTN binder shows equally high affinity to a ligand other than BTN. Based on DSC analysis the studied ligands did not differ significantly in their effect on the thermal stability of BBP-A and AVD (Table 2). Nor did the measured binding enthalpies vary significantly over ligand sets between AVD and BBP-A (Table 2). Overall, these ligand binding analyses suggest that all the studied ligands are efficiently associated (high negative binding enthalpy) with BBP-A and AVD. However, the determined dissociation rate of BTN from AVD was significantly lower than the dissociation rates of BSO and D-biotin sulfone, whereas the observed dissociation rates of the same ligands from BBP-A were rather similar to each other, the slowest observed for BSO. As a conclusion, it is the different dissociation rather than association rates that determines the ligand-binding preferences of BBP-A and AVD; out of the three ligands studied, AVD prefers BTN, whereas BBP-A seems to prefer BSO. Moreover, it is well known that the extremely slow dissociation of BTN from AVD is the main determinant of the high affinity binding [1].

One possible explanation for the differences in the dissociation rates of the studied ligands is the weaker structural stability of BBP-A as compared to AVD (Table 1). It has been experimentally shown, that binding of BTN to streptavidin lowers the rate of H/D exchange in large parts of the structure [10] at least partially due to the positive structural cooperativity in the binding process (thoroughly explained in [39]), *i.e. *improved packing and compactness of not only the ligand-binding site but of entire subunits, too. The weaker stability, or lower level of "compactness", of the entire BBP-A tetramer can therefore explain the differences in the observed ligand-binding properties of AVD and BBP-A. The BBP-A barrel fold may also be considered more flexible or dynamic than the AVD barrel, which could lead to a higher rate at which BTN is dislodged from the binding pocket, possibly by a mechanism similar to that presented by Hyre *et al. *for streptavidin [40].

What are the structural determinants that specify the recognition of BSO and why does the affinity of BSO meet that of BTN for BBP-A? The BBP-A – BSO structure is strikingly similar to the BBP-A – BTN structure – even the additional D-sulfoxide moiety of the ligand in the BBP-A – BSO structure does not seem to alter the binding pocket of BBP-A as compared to the BBP-A – BTN structure (Figure 6). One clear difference can, however, be detected: the hydrogen bond present in the BBP-A – BTN complex structure between Glu102 and BTN is missing in the BBP-A – BSO structure due to the different conformation of Glu102 in the two structures. Moreover, in the BBP-A – BSO structure, the carboxylate side-chain of Glu102 forms a salt-bridge with the guanidinium group of the Arg115 side-chain within the same subunit (in chains B and D) or is hydrogen bonded to a structural water molecule (in chains A and C), interactions that are not seen in the BBP-A – BTN complex. Arg115 is one of the conserved amino acids found at the 1–2 subunit interfaces of all known structures of members of the AVD family, but its conformation varies within the family (data not shown). This suggests that, in addition to the residues at the ligand-binding pocket, the type and conformation of residues at the subunit interfaces may regulate ligand binding, *e.g. *by allowing or disabling alternative hydrogen bonding networks.

## Conclusion

Our present study reveals that chicken has an avidin-like protein, BBP-A in its biotin-binding repertoire. Even though similar to AVD according to its three-dimensional structure and biotin-binding properties, *i.e. *the high affinity for BTN, BBP-A has several unique features – the thermal stability and immunological cross-reactivity of BBP-A is very different as compared to AVD. Furthermore, BBP-A is the first example of a protein from the AVD family that naturally has equally high affinity for BTN and for a non-BTN ligand. The biological function of BBP-A is not known, but since it binds both BSO and BTN with high affinity, it may have a function in the storage or delivery of BTN to an embryo as has been suggested in earlier studies for BBP-I and BBP-II [14,17]. In addition to providing clues of biological function and information about biotin-binding determinants in proteins, the high-resolution structure and biochemical analysis of BBP-A makes this novel protein attractive material for protein engineering and bio(nano)technological applications. For example, BBP-A could be covalently combined with AVD or AVR polypeptide using a circular permutation strategy [41,42] or used as a scaffold in the development of artificial enzymes [43].

## Methods

### Preparation of expression constructs

A cDNA clone of BBP-A [GenBank: BX930135] was obtained from the UK Chicken EST Consortium (ARK-Genomics). For insect cell production the cDNA, which included the sequence encoding for a putative signal peptide [18], was PCR amplified and cloned into a pFASTBAC1 vector (Invitrogen). The following primers were used: 5'BBP-A+signal (5'-AAA*AGATCT*ATGGAGCACCTCCGCTG) and 3'BBP-A (5'-ATTT*AAGCTT*ACTTGACACG GGTG), in which the restriction enzyme cleavage sites for BglII and HindIII, respectively, are shown in italics. In order to express BBP-A in *E. coli*, the original signal sequence of BBP-A was replaced with the OmpA signal peptide from *Bordetella avium *[44] by cloning into the pGemTeasy vector (Promega) containing the OmpA-signal and flanking attL recombination cloning sequences [30]. The construct was then transferred to the pBVboostFG expression vector [45,46] using LR recombination (Invitrogen). 5'BBP-A-core (5'-AAA*GGTACC*AGGAAGTGCGAGC; KpnI) and 3'BBP-A were used as the primers for PCR. The nucleotide sequences of the final expression vectors were confirmed by DNA sequencing.

The mutagenesis of *BBP-A *cloned in the pGemTeasy plasmid was performed using the QuikChange mutagenesis method (Stratagene, La Jolla, CA, USA). The *BBP-A *mutants were transferred to the pBVboostFG expression vector essentially as described previously [46].

### Protein expression and purification

BBP-A and its mutated forms were produced in *E. coli *BL-21(AI) cells (Invitrogen) as previously described [30]. The mutation A74S was created in order to study the significance of this residue for BTN binding (in AVD the side chain of the equivalent Ser73 forms a hydrogen bond with BTN). T118F, in turn, was created in order to increase the subunit contact area and consequently the stability of BBP-A; in AVD, I117Y was found to increase the stability of the protein [28]. iBBP-A, employing its natural signal peptide, was expressed in the eukaryotic host, *Spodoptera frugiperda*, using the Bac-to-Bac baculovirus expression system (Invitrogen). The proteins were isolated using 2-iminobiotin affinity chromatography (Affiland S. A., Liege, Belgium) as described elsewhere [30,47]. AVD isolated from chicken egg-white (wtAVD; Belovo S. A., Bastogne, Belgium) and AVD produced in *E. coli *(bAVD) [30] were used as control proteins throughout this study.

### Chemical synthesis of BSO and D-biotin sulfone

The syntheses of BSO and D-biotin sulfone were performed using a procedure described by Melville [48]. The melting points of the BTN derivatives were 201–202°C (lit. 200–203°C [48]) for BSO and 275–277°C (lit. 274–275°C [49]) for D-biotin sulfone. From ESI-MS analysis (see below) the following values were obtained: BSO, calculated molecular weight (C_10_H_16_N_2_O_4_S_1_) = 260.31, [M-H]^- ^*m*/*z *= 259.0753 and measured *m*/*z *= 259.0388; D-biotin sulfone, calculated molecular weight (C_10_H_16_N_2_O_5_S_1_) = 276.31, [M-H]^- ^*m*/*z *= 275.0702 and measured *m*/*z *= 275.0184.

### Stability analysis

The stability of BBP-A was analysed by SDS-PAGE as described previously [22]. Prior to analysis, the protein sample was acetylated *in vitro *and subsequently subjected to thermal treatment for 20 min in the presence of SDS and 2-mercaptoethanol. The oligomeric state of the treated protein was assessed by SDS-PAGE using Bio-Safe Coomassie (Bio-Rad) staining.

### Differential scanning calorimetry

Differential scanning analysis was performed in 50 mM NaPO_4 _buffer (pH 7.0) containing 100 mM NaCl as previously described [33]. The concentration of the analyzed proteins was between 0.2 and 0.6 mg/ml. In samples containing a ligand, the molar ligand concentration was three times as high as the protein subunit concentration. The samples were scanned from 25 to 130°C at a rate of 0.92°C/min.

### Gel filtration chromatography

Gel filtration analysis was performed with a ÄKTA™ purifier HPLC instrument (Amersham Biosciences) equipped with Superdex 200 10/300 GL column (Tricorn) as previously described [50]. A buffer containing 50 mM NaPO_4 _and 650 mM NaCl (pH 7.0) was used as the liquid phase. Biotin-complexed proteins were prepared by incubating the sample in the presence of 0.22 mM BTN 15 min prior to analysis.

### Mass spectrometry

The mass spectrometric studies were performed with a Micromass LCT ESI-TOF instrument equipped with a Z geometry electrospray ion source TOF detector. The analysis of BBP-A was performed as previously described [30]. Before analysis, the sample was dialysed against distilled water and lyophilised. MS analysis of the BTN used in the crystallization experiments with BBP-A was performed to determine whether BSO was present in the ligand solution. The amount of BSO in the sample was below the detection limit of the method (less than 1%). The detection limit was determined by making mixtures of 10 μg/ml (41 μM) of BTN with 1 (3.8 μM), 0.5 (1.9 μM) or 0.1 μg/ml (0.38 μM) of BSO.

### Ligand-binding analyses

The dissociation rate of [^3^H]biotin (Amersham) was measured at various temperatures with a competition assay as described previously [29]. Measurements were carried out in 50 mM NaPO_4 _buffer containing 100 mM NaCl.

The dissociation rate of the fluorescent biotin conjugate ArcDia™ BF560-biotin (ArcDia Ltd., Turku, Finland) was measured as reported previously [30]. The measurements were made at 25°C in 50 mM NaPO_4 _buffer (pH 7.0) containing 650 mM NaCl using a PerkinElmer LS55 luminometer.

The energetics of biotin binding was determined with a VP-ITC (MicroCal™) Isothermal Titration Calorimeter. Measurements were performed at 25°C in degassed 50 mM NaPO_4 _buffer (pH 7.0) containing 100 mM NaCl. In order to calculate the enthalpies of binding (ΔH) for BBP-A and AVD, the cell used for measurements was filled with 30 μM protein solution. BTN, BSO or D-biotin sulfone (0.5 mM) was then added to the measurement cell using 15 equal volume injections (10 μl) and at 240 second intervals. The data were analysed with Origin 7.0 software using the "One Set of Sites" method. The titration curve (heat change μcal/injection) resulting from the 15 injections was analysed by fitting the data to a nonlinear least square curve. We only determined the enthalpy of binding from these experiments, since the estimation of binding constants directly from the data was impossible due to the tight binding.

In order to study the intrinsic fluorescence of the proteins, the emission spectra of bBBP-A and wtAVD in 50 mM NaPO_4 _buffer (pH 7.0) containing 650 mM NaCl were measured using a PerkinElmer LS55 spectrofluorometer and excitation at 280 nm (slit 2.5 nm). During the analysis, protein solutions (100 nM) were continuously mixed using an integrated magnetic stirrer and maintained at 25°C using a circulating water bath. The emission spectra were also measured after addition of 200 nM ligand (BTN or its oxidised forms) to the protein solutions.

Fluorescence spectroscopy was also used to measure the rate of protein-ligand dissociation. These experiments were made at 40°C in order to measure the dissociation events within an experimentally applicable timescale. Firstly, the emission intensity of bBBP-A or wtAVD (50 nM) was measured at 350 nm (slit 10 nm) in the presence of BTN, BSO or D-biotin sulfone (100 nM). Secondly, in order to detect and quantify the dissociation events, a 1000-fold molar excess (100 μM) of BTN (BSO in case of determination of BTN dissociation) was added to the samples and the measurements were recorded for 3600 seconds. The measured spectral properties of each protein-ligand complex (*i.e. *the emission intensity of the protein-ligand complex) were used to create a single-phase dissociation model, which was fitted to the data. For example, in the case of the dissociation of BSO from BBP-A and binding of BTN to BBP-A, the dissociation process was observed as a decrease in the emission intensity (Figure 11c). The decrease in the fluorescence signal obtained in the control measurement performed in the presence of a 1000 molar excess of BTN was corrected during data analysis.

### Deglycosylation analysis

The BBP-A protein produced in insect cells was treated with Endoglycosidase H (New England Biolabs) to see if it was glycosylated. Wild-type AVD was used as a control protein. Prior to treatment, the analysed proteins were denatured by boiling them in the presence of 2-mercaptoethanol and SDS. Deglycosylation was performed overnight at 37°C. The samples were then boiled and subjected to SDS-PAGE analysis followed by staining with Bio-Safe Coomassie (Bio-Rad).

### Immunological analysis

The cross-reactivity of polyclonal AVD antibodies with BBP-A was studied using immunoblot and dot-blot analyses. The polyclonal rabbit antibodies TdaVIII [51] and an AVD antibody (University of Oulu, Finland) were used (dilution 1:5000) in Western blotting to analyse a 10 μg sample of BBP-A produced in *E. coli*. Three different amounts, 0.1, 1 and 10 μg of wtAVD, were used as controls in this experiment.

Dot-blot analysis was performed using only the TdaVIII antibody and 10 μg of BBP-A produced either in *E. coli *or in insect cells. AVD from chicken was used as a control (0.04–1.33 μg).

### Crystallization and diffraction data collection

Random and sparse matrix screens [52] prepared with the HamiltonSTAR robot in the Institute of Biotechnology at the University of Helsinki were initially used to search for suitable conditions for crystallization of BBP-A. Sitting drops of equal volumes (100 nl) of sample and well solution were automatically prepared by the Cartesian MicroSys robot on 96-well Greiner 3-SQ plates at 20°C. For optimization, the drop size was increased to 2 μl and crystallization was performed on conventional 24-well crystallization plates (Nextal/Hampton Research) using the vapour diffusion method and either sitting or hanging drops. In order to prepare BBP-A – BTN complexes, BTN (Sigma) diluted in buffer containing 5 mM Tris (pH 8.8) and 8 mM CHES (pH 9.5) was added to the protein samples in an approximate 1:10 molar ratio before crystallization. Two crystals were used to collect diffraction data and were obtained from conditions where 1 μl of protein solution (~0.4 mg/ml) containing 50 mM sodium acetate (pH 4.0) and 100 mM sodium chloride, and 1 μl of well solution containing either 2 M ammonium sulphate and 5% isopropanol (v/v) (BBP-A – BTN crystal) or 0.2 M sodium acetate, 0.1 M Tris (pH 8.6) and 30% (v/v) PEG 4000 (BBP-A – BSO crystal) were used. The diffraction data were collected at the MAX-lab beam line I711 (Lund, Sweden) at 100 K using a MarCCD detector. The BBP-A – BTN and BBP-A – BSO crystals were cryoprotected by adding 0.8 μl of 100% glycerol and 1 μl of 4 M sodium formate, respectively, to the crystallization drops just prior to flash-freezing in a 100 K liquid nitrogen stream (Oxford Cryosystem). Diffraction data were processed with programs of the XDS program package [53]. The data collection statistics are summarized in Table 3.

### Structure determination

The X-ray structures of BBP-A – BTN and BBP-A – BSO were solved using the molecular replacement program Amore [54] from the CCP4i suite [55,56]. A monomer of AVR2 [PDB: 1WBI] [24] was used as a trial model in Amore to solve the BBP-A – BTN structure. The BBP-A – BSO structure, in turn, was solved using the BBP-A – BTN structure as a search model in Amore. The best solutions from molecular replacement were selected as input for automatic model building with ARP/wARP [57]. The models were refined with Refmac5 [58], and modified and rebuilt with O [59]. Solvent atoms were added to the model with an automatic procedure in ARP/wARP [60] and other non-protein atoms were built manually in O. The coordinate file of the BSO ligand was obtained from the Cambridge Structural Database (CSD version 5.26) and molecular topologies of BSO were created with PRODGR [61]. The BBP-A structures were analyzed with the programs PROCHECK [62] and WHATIF [63]. The structure determination statistics are summarized in Table 3. The coordinates and structure factors of the BBP-A – BTN and BBP-A – BSO structures have been deposited in the Protein Data Bank with entry codes 2C1Q and 2C1S, respectively.

### Miscellaneous methods

Figures 2, 3, 4, 5 were created with the PyMOL Molecular Graphics System [64] and edited with the Corel Draw11 program suite. The multiple sequence alignment shown in Figure 2 was created using the program Malign implemented in BODIL [65]. Electrostatic potentials were calculated using the ABPS [66] plugin of PyMOL. The programs Contact and Areaimol [67] of the CCP4i suite were used to calculate the solvent accessible surface areas and to identify residues at the subunit interfaces, respectively.

Expressed sequence tag (EST) databases at NCBI were searched (blastn [32]) using the cDNA of BBP-A [GenBank: BX930135] and AVD [GenBank: X05343] as query sequences.

## Abbreviations

AVD, avidin; bAVD, AVD produced in bacteria; BBP, biotin-binding protein; bBBP-A, BBP-A expressed in bacteria; iBBP-A, BBP-A expressed in insect cells; BTN, D-biotin; BSO, D-biotin D-sulfoxide; DSC, differential scanning calorimetry; ESI-TOF, electrospray ionization time-of-flight; HABA, 4'-hydroxyazobenzene-2-carboxylic acid; ITC, isothermal titration calorimetry; wt, wild-type

## Authors' contributions

The biochemical studies of BBP-A and AVD were mainly carried out in the group of MSK at the Univ. of Jyväskylä and Tampere, whereas the structural analysis was mostly done in the groups of TAS and MSJ in the Structural Bioinformatics Laboratory, Åbo Akademi University. VPH carried out partially the protein expression and analysis experiments, JAEM and KKH carried out the calorimetric analyses, EAN was involved in the ligand-binding analyses and protein expression, JH synthesized BSO and D-biotin sulfone and carried out the mass spectrometric analyses, and KJH expressed and purified BBP-A and participated to the protein analyses. VPH, HRN, KR, OHL and MSK designed and supervised the experimental work related to biochemical analyses. TTA designed and carried out the 3D-structure analyses of BBP-A. VPH, MSJ, MSK, OHL and TTA were mainly responsible for writing and editing the manuscript. All authors read and approved the final manuscript.

## References

[B1] Green NM (1975). Avidin. Adv Prot Chem.

[B2] Botte V, Granata G (1977). Induction of avidin synthesis by RNA obtained from lizard oviducts. J Endocrinol.

[B3] Korpela JK, Kulomaa MS, Elo HA, Tuohimaa PJ (1981). Biotin-binding proteins in eggs of oviparous vertebrates. Experientia.

[B4] Laitinen OH, Hytönen VP, Ahlroth MK, Pentikäinen OT, Gallagher C, Nordlund HR, Ovod V, Marttila AT, Porkka E, Heino S, Johnson MS, Airenne KJ, Kulomaa MS (2002). Chicken avidin-related proteins show altered biotin-binding and physico-chemical properties as compared with avidin. Biochem J.

[B5] Green NM (1990). Avidin and streptavidin. Method Enzymol.

[B6] Bayer EA, Kulik T, Adar R, Wilchek M (1995). Close similarity among streptavidin-like, biotin-binding proteins from Streptomyces. Biochim Biophys Acta.

[B7] Nordlund HR, Hytönen VP, Laitinen OH, Kulomaa MS (2005). Novel avidin-like protein from a root nodule symbiotic bacterium, Bradyrhizobium japonicum. J Biol Chem.

[B8] Weber PC, Ohlendorf DH, Wendoloski JJ, Salemme FR (1989). Structural origins of high-affinity biotin binding to streptavidin. Science.

[B9] Livnah O, Bayer EA, Wilchek M, Sussman JL (1993). Three-dimensional structures of avidin and the avidin-biotin complex. Proc Natl Acad Sci USA.

[B10] Williams DH, Stephens E, Zhou M (2003). Ligand binding energy and catalytic efficiency from improved packing within receptors and enzymes. J Mol Biol.

[B11] Meslar HW, Camper SA, White HB (1978). Biotin-binding protein from egg yolk. A protein distinct from egg white avidin. J Biol Chem.

[B12] Murthy CV, Adiga PR (1984). Purification of biotin-binding protein from chicken egg yolk and comparison with avidin. Biochim Biophys Acta.

[B13] Subramanian N, Adiga PR (1995). Simultaneous purification of biotin-binding proteins-I and -II from chicken egg yolk and their characterization. Biochem J.

[B14] White HB, Whitehead CC (1987). Role of avidin and other biotin-binding proteins in the deposition and distribution of biotin in chicken eggs. Discovery of a new biotin-binding protein. Biochem J.

[B15] Bush L, McGahan TJ, White HB (1988). Purification and characterization of biotin-binding protein II from chicken oocytes. Biochem J.

[B16] Bush L, White HB (1989). Conversion of domains into subunits in the processing of egg yolk biotin-binding protein I. J Biol Chem.

[B17] White HB (1985). Biotin-binding proteins and biotin transport to oocytes. Ann N Y Acad Sci.

[B18] Niskanen EA, Hytönen VP, Grapputo A, Nordlund HR, Kulomaa MS, Laitinen OH (2005). Chicken genome analysis reveals novel genes encoding biotin-binding proteins related to avidin family. BMC Genomics.

[B19] Keinänen RA, Laukkanen ML, Kulomaa MS (1988). Molecular cloning of three structurally related genes for chicken avidin. J Steroid Biochem.

[B20] Keinänen RA, Wallén MJ, Kristo PA, Laukkanen MO, Toimela TA, Helenius MA, Kulomaa MS (1994). Molecular cloning and nucleotide sequence of chicken avidin-related genes 1-5. Eur J Biochem.

[B21] Ahlroth MK, Kola EH, Ewald D, Masabanda J, Sazanov A, Fries R, Kulomaa MS (2000). Characterization and chromosomal localization of the chicken avidin gene family. Anim Genet.

[B22] Bayer EA, Ehrlich-Rogozinski S, Wilchek M (1996). Sodium dodecyl sulfate-polyacrylamide gel electrophoretic method for assessing the quaternary state and comparative thermostability of avidin and streptavidin. Electrophoresis.

[B23] Pugliese L, Coda A, Malcovati M, Bolognesi M (1993). Three-dimensional structure of the tetragonal crystal form of egg-white avidin in its functional complex with biotin at 2.7 Å resolution. J Mol Biol.

[B24] Hytönen VP, Määttä JA, Kidron H, Halling KK, Hörhä J, Kulomaa T, Nyholm TK, Johnson MS, Salminen TA, Kulomaa MS, Airenne TT (2005). Avidin related protein 2 shows unique structural and functional features among the avidin protein family. BMC Biotechnol.

[B25] Eisenberg-Domovich Y, Hytönen VP, Wilchek M, Bayer EA, Kulomaa MS, Livnah O (2005). High-resolution crystal structure of an avidin-related protein: insight into high-affinity biotin binding and protein stability. Acta Crystallogr D.

[B26] DeTitta GT, Edmonds JW, Stallings W, Donohue J (1976). Molecular structure of biotin. Results of two independent crystal structure investigations. J Am Chem Soc.

[B27] Berman HM, Westbrook J, Feng Z, Gilliland G, Bhat TN, Weissig H, Shindyalov IN, Bourne PE (2000). The Protein Data Bank. Nucleic Acids Res.

[B28] Hytönen VP, Määttä JA, Nyholm TK, Livnah O, Eisenberg-Domovich Y, Hyre D, Nordlund HR, Hörhä J, Niskanen EA, Paldanius T, Kulomaa T, Porkka EJ, Stayton PS, Laitinen OH, Kulomaa MS (2005). Design and construction of highly stable, protease-resistant chimeric avidins. J Biol Chem.

[B29] Klumb LA, Chu V, Stayton PS (1998). Energetic roles of hydrogen bonds at the ureido oxygen binding pocket in the streptavidin-biotin complex. Biochemistry.

[B30] Hytönen VP, Laitinen OH, Airenne TT, Kidron H, Meltola NJ, Porkka E, Hörhä J, Paldanius T, Määttä JA, Nordlund HR, Johnson MS, Salminen TA, Airenne KJ, Ylä-Herttuala S, Kulomaa MS (2004). Efficient production of active chicken avidin using a bacterial signal peptide in Escherichia coli. Biochem J.

[B31] Edwards K, Chan RY, Sawyer WH (1994). Interactions between fatty acids and lipoprotein lipase: specific binding and complex formation. Biochemistry.

[B32] NCBI blastn. http://www.ncbi.nlm.nih.gov/blast/.

[B33] Hytönen VP, Nyholm TK, Pentikäinen OT, Vaarno J, Porkka EJ, Nordlund HR, Johnson MS, Slotte JP, Laitinen OH, Kulomaa MS (2004). Chicken avidin-related protein 4/5 shows superior thermal stability when compared with avidin while retaining high affinity to biotin. J Biol Chem.

[B34] Flower DR (1996). The lipocalin protein family: structure and function. Biochem J.

[B35] White HB, Dennison BA, Della Fera MA, Whitney CJ, McGuire JC, Meslar HW, Sammelwitz PH (1976). Biotin-binding protein from chicken egg yolk. Assay and relationship to egg-white avidin. Biochem J.

[B36] Mandella RD, Meslar HW, White HB (1978). Relationship between biotin-binding proteins from chicken plasma and egg yolk. Biochem J.

[B37] Seshagiri PB, Adiga PR (1987). Identification and molecular characterisation of a biotin-binding protein distinct from avidin of chicken egg white and comparison with yolk biotin-binding protein. Biochim Biophys Acta.

[B38] Melville DB, Genghof DS, Lee JM (1954). Biological properties of biotin d- and l-sulfoxides. J Biol Chem.

[B39] Williams DH, Stephens E, O'Brien DP, Zhou M (2004). Understanding noncovalent Interactions: Ligand binding energy and catalytic efficiency from ligand-induced reductions in motion within receptors and enzymes. Angew Chem Int Ed.

[B40] Hyre DE, Amon LM, Penzotti JE, Le Trong I, Stenkamp RE, Lybrand TP, Stayton PS (2002). Early mechanistic events in biotin dissociation from streptavidin. Nat Struct Biol.

[B41] Nordlund HR, Laitinen OH, Hytönen VP, Uotila ST, Porkka E, Kulomaa MS (2004). Construction of a dual chain pseudotetrameric chicken avidin by combining two circularly permuted avidins. J Biol Chem.

[B42] Hytönen VP, Nordlund HR, Hörhä J, Nyholm TK, Hyre DE, Kulomaa T, Porkka EJ, Marttila AT, Stayton PS, Laitinen OH, Kulomaa MS (2005). Dual-affinity avidin molecules. Proteins.

[B43] Thomas CM, Ward TR (2005). Artificial metalloenzymes: proteins as hosts for enantioselective catalysis. Chem Soc Rev.

[B44] Gentry-Weeks CR, Hultsch AL, Kelly SM, Keith JM, Curtiss R (1992). Cloning and sequencing of a gene encoding a 21-kilodalton outer membrane protein from Bordetella avium and expression of the gene in Salmonella typhimurium. J Bacteriol.

[B45] Airenne KJ, Peltomaa E, Hytönen VP, Laitinen OH, Ylä-Herttuala S (2003). Improved generation of recombinant baculovirus genomes in Escherichia coli. Nucleic Acids Res.

[B46] Laitinen OH, Airenne KJ, Hytönen VP, Peltomaa E, Mähönen AJ, Wirth T, Lind MM, Mäkelä KA, Toivanen PI, Schenkwein D, Heikura T, Nordlund HR, Kulomaa MS, Ylä-Herttuala S (2005). A multipurpose vector system for the screening of libraries in bacteria, insect and mammalian cells and expression in vivo. Nucleic Acids Res.

[B47] Airenne KJ, Oker-Blom C, Marjomäki VS, Bayer EA, Wilchek M, Kulomaa MS (1997). Production of biologically active recombinant avidin in baculovirus-infected insect cells. Prot Exp Pur.

[B48] Melville DB (1954). Biotin sulfoxide. J Biol Chem.

[B49] Hofmann K, Melville DB, du Vigneaud V (1941). Characterization of the functional groups of biotin. J Biol Chem.

[B50] Nordlund HR, Hytönen VP, Laitinen OH, Uotila ST, Niskanen EA, Savolainen J, Porkka E, Kulomaa MS (2003). Introduction of histidine residues into avidin subunit interfaces allows pH-dependent regulation of quaternary structure and biotin binding. FEBS Lett.

[B51] Kulomaa MS, Elo HA, Tuohimaa PJ (1978). A radioimmunoassay for chicken avidin. Comparison with a [14C]biotin-binding method. Biochem J.

[B52] Jancarik J, Scott WG, Milligan DL, Koshland DE, Kim SH (1991). Crystallization and preliminary X-ray diffraction study of the ligand-binding domain of the bacterial chemotaxis-mediating aspartate receptor of Salmonella typhimurium. J Mol Biol.

[B53] Kabsch W (1993). Automatic Processing of Rotation Diffraction Data from Crystals of Initially Unknown Symmetry and Cell Constants. J Appl Crystallogr.

[B54] Navaza J (1994). Amore - an automated package for molecular replacement. Acta Crystallogr A.

[B55] Collaborative computational project, number 4 (1994). The CCP4 suite: programs for protein crystallography.. Acta Crystallogr D.

[B56] Potterton E, Briggs P, Turkenburg M, Dodson E (2003). A graphical user interface to the CCP4 program suite. Acta Crystallogr D.

[B57] Perrakis A, Morris R, Lamzin VS (1999). Automated protein model building combined with iterative structure refinement. Nat Struct Biol.

[B58] Murshudov GN, Vagin AA, Dodson EJ (1997). Refinement of macromolecular structures by the maximum-likelihood method. Acta Crystallogr D.

[B59] Jones TA, Zou JY, Cowan SW, Kjeldgaard (1991). Improved methods for building protein models in electron density maps and the location of errors in these models. Acta Crystallogr A.

[B60] Lamzin VS, Wilson KS (1993). Automated refinement of protein models. Acta Crystallogr D.

[B61] Schuttelkopf AW, van Aalten DM (2004). PRODRG: a tool for high-throughput crystallography of protein-ligand complexes. Acta Crystallogr D.

[B62] Laskowski RA, Macarthur MW, Moss DS, Thornton JM (1993). Procheck - a program to check the stereochemical quality of protein structures. J Appl Crystallogr.

[B63] Vriend G (1990). WHAT IF: a molecular modeling and drug design program. J Mol Graph.

[B64] DeLano WL The PyMOL Molecular Graphics System. http://pymol.sourceforge.net/.

[B65] Lehtonen JV, Still DJ, Rantanen VV, Ekholm J, Björklund D, Iftikhar Z, Huhtala M, Repo S, Jussila A, Jaakkola J, Pentikäinen OT, Nyrönen T, Salminen TA, Gyllenberg M, Johnson MS (2004). BODIL: a molecular modeling environment for structure-function analysis and drug design.. J Comput Aided Mol Des.

[B66] Baker NA, Sept D, Joseph S, Holst MJ, McCammon JA (2001). Electrostatics of nanosystems: application to microtubules and the ribosome. Proc Natl Acad Sci USA.

[B67] Lee B, Richards FM (1971). The interpretation of protein structures: estimation of static accessibility. J Mol Biol.

[B68] Repo S, Paldanius TA, Hytönen VP, Nyholm TK, Halling KK, Huuskonen J, Pentikäinen OT, Rissanen K, Slotte JP, Airenne TT, Salminen TA, Kulomaa MS, Johnson MS (2006). Binding properties of HABA-type azo derivatives to avidin and avidin-related protein 4. Chem Biol.

